# Uplink OFDM detection with random multiple access

**DOI:** 10.1038/s41598-022-13956-x

**Published:** 2022-06-21

**Authors:** Amir J. Salomon, Benjamin G. Salomon, Ofer Amrani

**Affiliations:** 1 Department of Electrical Engineering-Systems, Tel Aviv University, 69978 Ramat Aviv, Tel-Aviv Israel; 2 Faculty of Engineering, Ruppin Academic Center, 40250 Emek Hefer, Israel

**Keywords:** Electrical and electronic engineering, Applied mathematics

## Abstract

Orthogonal Frequency-Division Multiplexing with Random multiple access (OFDRMA) is discussed for uplink communications, whereby several active users send information towards a single base-station (BS), while all other users are dormant. Originally, uplink communication methods included sharing the frequency resources among the active users in an orthogonal fashion, i.e., a central unit is required to dynamically allocate the resources. More recently, non-orthogonal methods have arisen, meaning that several active users share the same frequency bins, but they still do require a central unit to dynamically allocate the resources in a uniform (as possible) manner over the available bandwidth. The task and overhead required for managing the frequency allocations among the users can be quite cumbersome. In OFDRMA, the frequency allocations for any user are independent of the frequency allocations for the other users, and independent of which of the other users are currently active. Rather, OFDRMA relies on random, yet predetermined, allocation of frequency bins for each user, known only to that user and the BS. A multi-user detection approach is presented based on a graphical representation of the system. It is shown to provide robustness against the forced randomness of the scheme. Capacity of OFDRMA and its optimization are analyzed and provided in detail. Simulation results are provided for demonstrating the performance attainable with OFDRMA and the proposed detection scheme. Both the capacity and the simulations are compared with modern multi-user multiple-input multiple-output (MU-MIMO) schemes.

## Introduction

Orthogonal frequency-division multiplexing (OFDM) is nowadays a common practice for data transmission, where the binary information is first modulated into a larger symbol constellation, and the modulated sequences are transmitted over multiple carrier frequencies. The total available bandwidth is divided into units called frequency subcarriers (a.k.a. bins). Each bin is used to transmit a separate narrow-band signal. A major advantage of OFDM compared to single-carrier schemes is that it dose not requite complex equalization in order to provide reliable communications.

In a typical multiuser environment incorporating OFDM, a number of users employ the same transmission medium over the same frequency band to transmit simultaneously towards a single BS. In order to minimize interference among the active users, the BS dynamically allocates to each active user a set of bins, over which the user and the BS convey information between each other. Ideally, this set of bins is disjoint from the bins employed by all other transmitting users, but it is not always possible to achieve that.

The process of orchestrating the frequency allocations among the users can be quite cumbersome in certain scenarios, and even impossible in others. For example, consider a network cell in which the user equipment must be simple, where no channel state information (CSI) is assumed at the users’ side and the channel is frequency selective. This may very well be the case for certain IoT (Internet of Things)^1^, smart grid applications, and certain power-line communication (PLC) systems.

Another relevant scenario can be encountered when an OFDMA (OFDM with multiple access) network controller ceases to operate. The network controller is typically a part of the BS; it periodically sends short messages to all the active users using the downlink channel, and receives short messages from all the active users on the uplink channel. These type of control messages on the uplink channel originate from the users, and they inform the network controller that a user becomes active or becomes inactive accordingly. The network controller analyzes these messages and takes note of the total number of active users. Then, the network controller informs each of these users, by sending control downlink messages, which subcarriers it should use in the near future for transmitting data towards the BS. The network controller tries to allocate the users over the frequency bins as uniformly as possible, i.e., that each bin will contain data from the the same number of active users, or at least as close as possible. When the network controller cease to operate, some other mechanism for dynamically allocating the frequency resources between the users should be activated.

To handle the scenarios above, among others, we will develop a new approach, which incorporates (pseudo) randomness into the OFDMA-based scheme. In the absence of uplink channel-knowledge at the users’ side, and lack of specific resource allocation guidance from the BS, each user shall occupy a unique (pseudo) random set of bins known (only) to that user and the BS. The set of bins employed by each user is fixed (much like an identifying medium access control (MAC) address). A table (code-book) holding the complete bin-usage for all the users in the network will be stored at the BS. We refer to the obtained scheme as OFD**R**MA (Orthogonal Frequency Division with **R**andom Multiple Access). OFDRMA may be considered as an instance of non-orthogonal multiple access (NOMA). NOMA has been discussed for downlink channel^2,3^ with random access, to which one can refer as NORMA. NOMA for wireless networks uplink channel has been discussed^4^ with deterministic (”non-random”) resource allocation; the interference among active users is controlled and restrained by a central controller, hence retaining the overhead and complexity associated with orchestrating the frequency resource allocation. Uplink NOMA for 5G systems using uplink power control has been discussed^5,6^, aiming at optimizing the outage performance and the achievable sum data rate. User pairing and power allocation in NOMA has been discussed for both the uplink channel^7,8^ and the downlink channel^6,8^. Power domain NOMA has been discussed for both the uplink and the downlink channels^9^, where the BS controls the power and rate of all the users according to the channel state information.

Random usage of frequency resources was considered in the late 90’s by Nogueroles et. al.^10^, where a few basic aspects of the scheme were briefly studied theoretically. Employing OFDRMA for the, so-called, ”disaster recovery” scenario has been described in detail^11^. The concept of OFDRMA in the downlink transmission channel has been studied recently by the authors of this paper^12^. The concept of OFDRMA in the uplink transmission channel alongside an effective multi-user detection algorithm has not been studied or evaluated in the past.

In OFDRMA collisions may occur when two users, or more, simultaneously occupy the same bin. Clearly, the number and pattern of collisions depend on the number of active users, message length, modulation order, number of available bins, and the required bit rate. In this paper, to effectively mitigate the influence of collisions and recover the information reliably, error-control coding is introduced at the transmitter side, and a specific multi-user detection (MUD) algorithm is proposed to be carried out at the BS side. The combined MUD and error correction decoding algorithms proposed herein are shown to provide robustness against the collisions even under severe collision scenarios. The rest of the paper is organized as follows. Background and general system description are given in Sec. 2. Basic properties of collisions’ behavior are briefly discussed in the Sec. 3. A graphical representation of the detection scheme is discussed briefly in Sec. 4. A first detection algorithm is presented in Sec. 5; this algorithm employs maximum a posteriori (MAP) estimation method. In Sec. 6 a second detection algorithm is presented, which employs a reduced complexity suboptimal linear estimation method. Sec. 7 provides detailed analysis of the OFDRMA capacity and how to maximize the capacity of any given OFDRMA layout. OFDRMA capacity is thoroughly compared with the capacity of corresponding MU-MIMO schemes. Sec. 8 provides simulation results for some scenarios. The simulation results are thoroughly compared with simulation results of corresponding MU-MIMO schemes. Finally, conclusions are drawn in Sec. 9.

## Preliminaries

Herein, we consider a ”single-cell” environment, i.e., a single access-point (base station), serving *u* active users. *u* is effectively a random variable (RV), as will be discussed later, assumed to be known to the BS (The algorithm by which *u* becomes known to the BS is outside the scope of this work). Each user is assumed to have a single transmit antenna, but this can be easily generalized. The number of receive antennas at the BS is denoted by $\vartheta$.

A transmission by any (active) user is divided into units called *packets*. The total number of frequency subcarriers, available for information transmission in each packet, is denoted by *m*. *m* is termed the *packet length*; typical values for *m* range between 100–10, 000. The frequency-domain intercepted signals on the BS side are denoted by $F^a_l$, with *l* being the subcarrier index number, $1 \le l \le m$, and *a* being the index number of the receive antenna, $1 \le a \le \vartheta$.

Let ${\bar{X}}_i$ denote a length-*n* coded and modulated sequence that user *i* transmits toward the BS. Correspondingly, *n* is the number of frequency bins user *i* employs in a single packet (we assume that *n* is the same for all users, but this can be easily generalized), where $n \le m$.

The coded and modulated sequence ${\bar{X}}_i$ is given by:

1 $$\begin{aligned} {\bar{X}}_i = [ X_{i1} , X_{i2} , \ldots , X_{in} ] . \end{aligned}$$

Each of the elements of ${\bar{X}}_i$ is chosen from a pre-defined finite constellation. Binary error-correcting codewords are generated by each user and mapped onto constellation points. For example, a user shall employ a single codeword per packet when using BPSK modulation, and two codewords per packet when QPSK modulation is employed.

In the following we assume that each user employs QPSK for the sake of notational brevity; transmission using BPSK constellation is straightforwardly derived by simplifying the following expressions, and the development for higher order constellations is quite similar.

When using QPSK, 2 bits select a constellation point. Therefore, in each transmission, each active user generates two binary codewords:

2 $$\begin{aligned} {\bar{x}}^1_i= & {} [ x^1_{i1}, x^1_{i2}, \ldots , x^1_{in} ] , \end{aligned}$$

3 $$\begin{aligned} {\bar{x}}^2_i= & {} [ x^2_{i1}, x^2_{i2}, \ldots , x^2_{in} ] , \end{aligned}$$

belonging to the binary, length-*n*, codes ${\mathcal {C}}^1$, ${\mathcal {C}}^2$, ${\bar{x}}^1_i \subset {\mathcal {C}}^1$, ${\bar{x}}^2_i \subset {\mathcal {C}}^2$. Typically, ${\mathcal {C}}^1$ and ${\mathcal {C}}^2$ are permutated versions of each other^13^. Each bit-pair is then mapped to a constellation point:

4 $$\begin{aligned} X_{ij} = \mathbb {S}_{x^1_{ij} x^2_{ij}}. \end{aligned}$$

A QPSK point in the complex signal space is given by:

5 $$\begin{aligned} \mathbb {S}_{x^1_{ij} x^2_{ij}}=\sqrt{\frac{\omega }{2}} \cdot \left[ (-1)^{x^1_{ij}} + \iota \cdot (-1)^{x^2_{ij}} \right] , \end{aligned}$$

where $\omega$ is the mean signal energy:

6 $$\begin{aligned} \omega = E \left( \Vert \mathbb {S}_{x^1_{ij} x^2_{ij}} \Vert ^2 \right) . \end{aligned}$$

and $\iota$ is the imaginary unit $\iota ^2 = -1$.

The BS holds the following information concerning all the users operating within its cell:

### Definition 1

The allocation vector of user $i, 1 \le i \le u$, denoted by:

$$\begin{aligned} {\bar{t}}_i = [ t_{i1} , t_{i2} , \ldots , t_{in} ], \end{aligned}$$

is a size *n* (pseudo) random subset of $\{ 1 , 2 , \ldots \ m \}$ ; each $t_{ij}$ denotes the frequency bin on which the $i^{th}$ user sends its $j^{th}$ symbol. Each user *i* knows only its own ${\bar{t}}_i$, but the BS knows all ${\bar{t}}_i , 1 \le i \le u$.

The signals received at the BS receiver on frequency subcarrier *l* are affected by all the users transmitting on this subcarrier. Using OFDM frequency-domain equivalent channel, we get:

7 $$\begin{aligned} F^a_l = \sum _{i=1}^{u} \sum _{j=1}^{n} I(l=t_{ij}) \cdot X_{ij} \cdot h^a_{il} + n^a_l \end{aligned}$$

where $I(\cdot )$ is the indicator function and $h^a_{il}$ is the path gain on subcarrier *l* between user *i* and receive antenna *a*. The path gains are mutually independent and identically distributed (i.i.d) circularly-symmetric complex normal RVs with variance of 1 per sample. The values of $h^a_{il}$ are assumed to be known only to the BS (the algorithms by which the path gains become known to the BS are outside the scope of this work^14^). The noise samples $n^a_l$ are i.i.d circularly-symmetric complex normal RVs with variance $\sigma ^2$ per sample.

## Collision events

Collisions occur when at least two users transmit simultaneously on the same subcarrier.

### Definition 2

Let $c_l$ be the number of (non-zero) terms included in the double sum (7) of $F_l$ for a specific bin *l* (i.e., the number of users contributing to the value of $F_l$). If $c_l > 1$, we say that collision occurred on the $l^{th}$ subcarrier, with $c_l$ being the order of the collision.

Collision events may degrade system performance in terms of increased error rate, but no less important is the increased decoding complexity at the BS. First, we briefly derive a simple mathematical model for collision events.

The first user distributes *n* symbols in *m* distinct bins selected randomly. The event that a specific bin was chosen has a Bernoulli distribution with probability:

8 $$\begin{aligned} p=\frac{n}{m} \end{aligned}$$

The parameter *p*, which may be either fixed or viewed as an utilization factor, is of particular importance later in the paper, where we analyze the OFDRMA scheme capacity and its optimization.

The second user repeats the same process independently from the first user. For *u* active users, the number of users transmitting (colliding) on a specific bin has a Binomial distribution, the probability for collision of order *c* on any bin is:

9 $$\begin{aligned} p(c_l = c) = \left( {\begin{array}{c}u\\ c\end{array}}\right) \cdot \left( \frac{n}{m} \right) ^c \cdot \left( 1-\frac{n}{m} \right) ^{u-c}. \end{aligned}$$

The average number of collisions on a single bin is:

10 $$\begin{aligned} E(c_l) = \frac{u \cdot n}{m} , \end{aligned}$$

and we can also state that:

11 $$\begin{aligned} \sum ^{m}_{l=1} c_l = u \cdot n , \end{aligned}$$

as both sides of the equation are equal to the total number of interceptions at the BS, for all the users transmitting within the same packet.

It should be noted that in practice, the users have fixed allocation vectors; however, since these vectors are generated randomly, and the *u* active users are taken (at random) from a much larger number of possible users, the above discussion is statistically correct.

It is evident from the above discussions that in order to minimize the occurrence of collisions, a small number of users, *u*, is desired (practically not controllable), and a large number of available frequency bins, *m*, is desired - which in turn reduces the transmission rate and increases latency. Shorter code length, *n*, also reduces the occurrence of collisions, but using shorter error correcting codes typically implies higher decoding error rate.

## Graphical representation of the detection scheme

**Figure 1:**
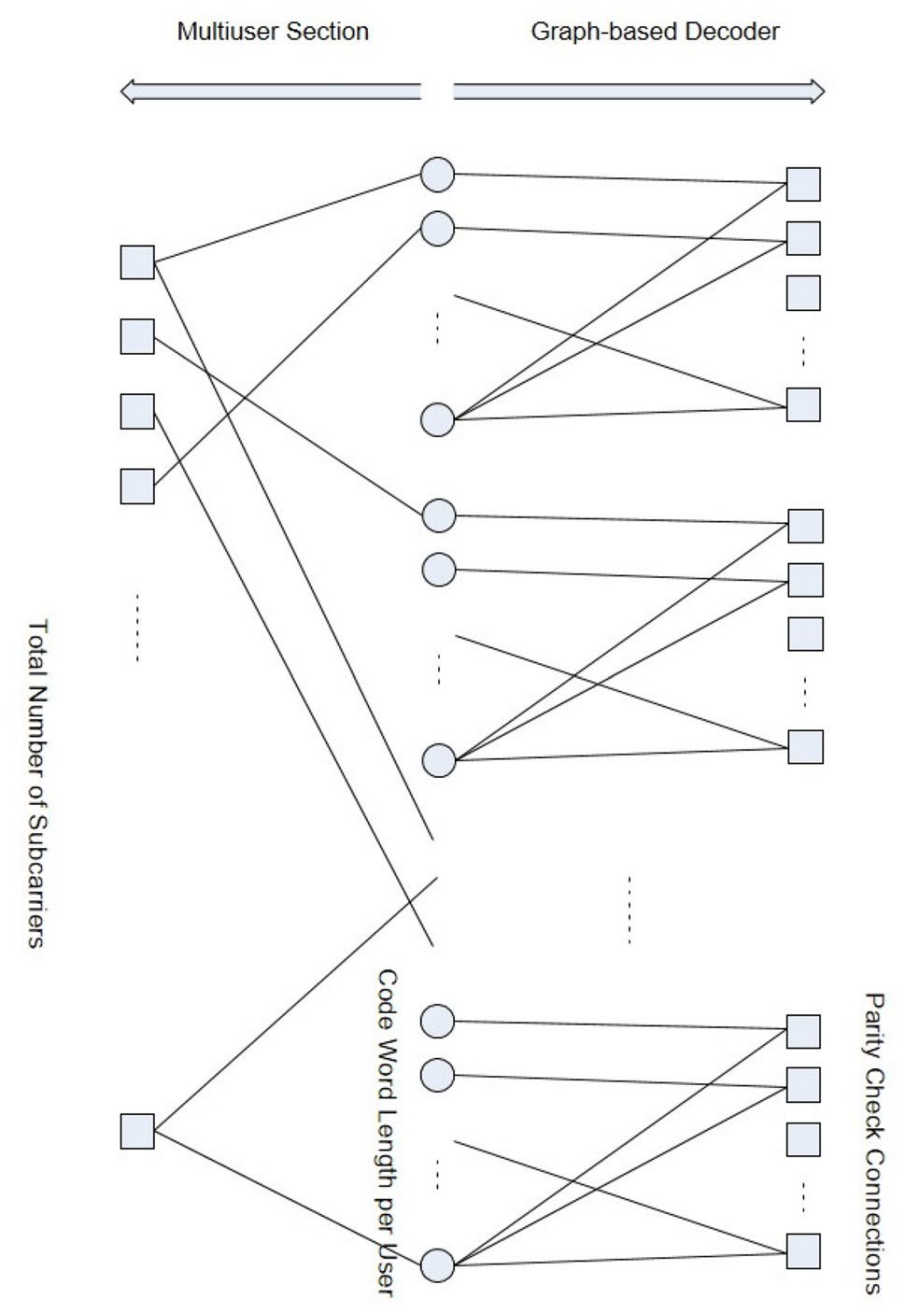
OFDRMA unified graphical representation.

A unified graphical representation for the complete frequency allocation and coding scheme that captures all the active users is natural for OFDRMA. The graph can be divided into two interconnected bi-partite subgraphs as depicted in Fig. 1. The coded bits (denoted by circles), placed in the middle, connect the two sides of the graph.

The left-hand-side (LHS) subgraph represents the bits (circles) and their allocation (LHS edges) among all the available frequency bins (denoted by the LHS squares). In other words, the modulated symbol that carries a certain bit is transmitted on a certain bin if an edge connects the corresponding circle and square. If more than one edge is connected to a (LHS) square, it means that multiple users share the same frequency bin, leading to a collision on that bin. The number of edges coming out of this square is the *collision order*.

The right-hand-side (RHS) subgraph represents the error-correcting mechanism of this scheme. It is composed of a multiplicity of disjoint (bi-partite) graphs associated with the error-correcting parity check matrices of the active users. Each parity-check equation (RHS squares) connects (via RHS edges) to an appropriate set of bits (circles). Typically, this would imply that *u* copies of the same diagram are placed vertically, assuming each user employs the same error correcting code. This situation is demonstrated in Fig. 1. However, in general, this is not necessarily the case as each user can use a different code, if required.

The decoding process amounts to messages passing along the graph edges from left to right and vice versa. Initial bit reliability messages are extracted from the frequency bins and passed to the bits over the LHS edges. Then, messages iterate back and forth on the RHS edges for a desired number of iterations. Then, messages pass left and right (once) along the LHS edges, in order to update the initial bit reliability messages according to the other bits it shares on the same frequency bin. This whole process is repeated for a few cycles, as required. This iterative detection process is detailed in the following sections.

## Multi-user detection using maximum a posteriori approach

Assume that each user transmits two LDPC codewords in a single packet using QPSK modulation (5). For the $i^{th}$ user, we need to calculate the probability (12) of the $j^{th}$ bit of the first code $x^1_{ij}$ (the calculation for the corresponding second codeword bit $x^2_{ij}$ is similar). First, the receiver (BS) uses the vector ${\bar{t}}_i$ to find the frequency bin, denoted by $\alpha$, over which this bit was transmitted, i.e. $\alpha =t_{ij}$. We list all the other users that transmit on the same bin and their corresponding bit indexes by the following set:

$$\begin{aligned} {\mathbb {D}}^\alpha _i = \bigcup _{t_{{\tilde{i}},{\tilde{j}}}=\alpha } \{ {\tilde{i}},{\tilde{j}} \}, \qquad 1 \le {\tilde{i}} \ne i \le u , 1 \le {\tilde{j}} \le n \end{aligned}$$

${\mathbb {D}}^\alpha _i$ is a (vector) set consisting of pairs of integer numbers; each pair indicates a user number and its corresponding bit index transmitted over frequency bin $\alpha$.

The order of the collision occurring in bin $\alpha$ is denoted by $c_{\alpha }$, as discussed in Sec. 3. The number of elements (pairs) in each set ${\mathbb {D}}^\alpha _i$ is evidently $c_{\alpha } - 1$. For simplicity of notation and without loss of generality, we replace $c_{\alpha }$ with *c*, we assume that $i=1$, and assume that users numbered $2 , 3 , \ldots , c$ have collided with user 1 in bin $\alpha$. The bit indexes of the corresponding codewords are marked by $\xi _e$, $1 \le e \le c$ (for instance, $\xi _1 = j$, as implied by the description above).

During the decoding process, the BS stores and periodically updates 2 tables; one table for each of the 2 error-correcting codewords (as we assume QPSK modulation). The size of each table is $u \times n$, as it stores the current probabilities for all the bits associated with all the users:

12 $$\begin{aligned} p^1_{ij}=prob(x^1_{ij}=1) \,\,\,\,\, , \,\,\,\,\, p^2_{ij}=prob(x^2_{ij}=1) , \end{aligned}$$

where *i* is the user number indicator, $1 \le i \le u$, and *j* is the bit index indicator, $1 \le j \le n$.

For brevity of notation in the expressions that follow, we replace the sum notation

$$\begin{aligned} \sum _{x^2_{1 \xi _1}, x^1_{2 \xi _2}, x^2_{2 \xi _2}, \cdots , x^1_{e \xi _e}, x^2_{e \xi _e}, \cdots , x^1_{c \xi _c}, x^2_{c \xi _c}} \end{aligned}$$

simply by $\underset{x}{\sum }$ for the discussion that follows.

We need to calculate the probability for $x^1_{1j}=\beta$, $\beta \in \{0,1\}$; this probability is affected directly by all the signal values intercepted on the $\vartheta$ BS receive antennas on bin $\alpha$, which are $F^1_{\alpha }, F^2_{\alpha }, \ldots , F^\vartheta _{\alpha }$ :

13 $$\begin{aligned}&p(x^1_{1j}=\beta |F^1_{\alpha }, F^2_{\alpha }, \ldots , F^\vartheta _{\alpha }) = \frac{f(F^1_{\alpha }, \ldots , F^\vartheta _{\alpha }|x^1_{1j}=\beta ) \cdot p(x^1_{1j}=\beta )}{f(F^1_{\alpha }, \ldots , F^\vartheta _{\alpha })} \underset{(a)} = \,\,\,\,\,\,\,\,\,\, \nonumber \\&\quad \sum _x f \left( F^1_{\alpha }, \ldots , F^\vartheta _{\alpha }|x^1_{1j}=\beta , x^2_{1 \xi _1}, x^1_{2 \xi _2}, x^2_{2 \xi _2}, \cdots , x^1_{e \xi _e}, x^2_{e \xi _e}, \cdots , x^1_{c \xi _c}, x^2_{c \xi _c} \right) \nonumber \\&\quad \cdot p(x^2_{1 \xi _1}, x^1_{2 \xi _2}, x^2_{2 \xi _2}, \cdots , x^1_{e \xi _e}, x^2_{e \xi _e}, \cdots , x^1_{c \xi _c}, x^2_{c \xi _c}) \cdot \frac{0.5}{f(F^1_{\alpha }, \ldots , F^\vartheta _{\alpha })} = \nonumber \\&\quad \sum _x \prod ^\vartheta _{d=1} f \left( F^d_{\alpha }|x^1_{1j}=\beta , x^2_{1 \xi _1}, x^1_{2 \xi _2}, x^2_{2 \xi _2}, \cdots , x^1_{e \xi _e}, x^2_{e \xi _e}, \cdots , x^1_{c \xi _c}, x^2_{c \xi _c} \right) \nonumber \\&\quad \cdot p(x^2_{1 \xi _1}, x^1_{2 \xi _2}, x^2_{2 \xi _2}, \cdots , x^1_{e \xi _e}, x^2_{e \xi _e}, \cdots , x^1_{c \xi _c}, x^2_{c \xi _c}) \cdot \frac{0.5}{f(F^1_{\alpha }, \ldots , F^\vartheta _{\alpha })} \underset{(b)}= \nonumber \\&\quad \sum _x \prod ^\vartheta _{d=1} \exp {\left\{ -\frac{1}{\sigma ^2} \cdot \left| F^d_{\alpha }-S_{\beta x^2_{1 \xi _1}} \cdot h^d_{1 \alpha }-\sum _{e=2}^c S_{x^1_{e \xi _e}, x^2_{e \xi _e}} \cdot h^d_{e \alpha } \right| ^2 \right\} } \nonumber \\&\quad \cdot p(x^2_{1 \xi _1})p(x^1_{2 \xi _2})p(x^2_{2 \xi _2}) \cdots p(x^1_{e \xi _e})p(x^2_{e \xi _e}) \cdots p(x^1_{c \xi _c})p(x^2_{c \xi _c}) \cdot \frac{0.5}{(\pi \sigma ^2)^\vartheta \cdot f(F^1_{\alpha }, \ldots , F^\vartheta _{\alpha })} = \nonumber \\&\quad \sum _x \exp {\left\{ -\frac{1}{\sigma ^2} \cdot \sum ^\vartheta _{d=1} \left| F^d_{\alpha }-S_{\beta x^2_{1 \xi _1}} \cdot h^d_{1 \alpha }-\sum _{e=2}^c S_{x^1_{e \xi _e}, x^2_{e \xi _e}} \cdot h^d_{e \alpha } \right| ^2 \right\} } \nonumber \\&\quad \cdot \prod ^c_{e=2} (p^1_{e \xi _e})^{x^1_{e \xi _e}} (1-p^1_{e \xi _e})^{1-x^1_{e \xi _e}} \cdot \prod ^c_{e=1} (p^2_{e \xi _e})^{x^2_{e \xi _e}} (1-p^2_{e \xi _e})^{1-x^2_{e \xi _e}} \cdot \frac{0.5}{(\pi \sigma ^2)^\vartheta \cdot f(F^1_{\alpha }, \ldots , F^\vartheta _{\alpha })}, \end{aligned}$$

where $f(F^1_{\alpha }, \ldots , F^c_{\alpha })$ is the joint probability density function of the values intercepted on all the receive antennas on bin $\alpha$; we do not need to explicitly calculate this probability, as the decoding process requires only the ratio

$$\begin{aligned} \frac{p(x^1_{1j}=0|F^1_{\alpha }, F^2_{\alpha }, \ldots , F^\vartheta _{\alpha })}{p(x^1_{1j}=1|F^1_{\alpha }, F^2_{\alpha }, \ldots , F^\vartheta _{\alpha })} , \end{aligned}$$

hence the term $f(F^1_{\alpha }, \ldots , F^c_{\alpha })$ is cancelled out in both the numerator and the denominator.

Remarks to (13):

- We take a-priori value $p(x^1_{1j}=1)=0.5$ always instead of the current $p^1_{1j}$ value in order to minimize circulation of the same information on the code graphs (similar to the concept of minimizing cycles in LDPC codes^15^); all the other probability values are taken from the probability tables (12) that are updated periodically.
- The bit values are initially i.i.d., however as the algorithm progresses, some dependency is introduced due to the passing of information between frequency bins, receive antennas and distinct codeword bits; however, the i.i.d. assumption proves to be sufficiently accurate.

In view of the above derivation, a multi-user detection (MUD) algorithm can be stated as follows:

### Algorithm 1

1. Initialize the probability matrices $p^1_{ij}=p^2_{ij}=0.5$, $1 \le i \le u$, $1 \le j \le n$.
2. Update the probability matrices according to (13).
3. Compute the log-likelihood ratio (LLR) metrics for each bit of each user:
  14 $$\begin{aligned} llr^1_{ij} = \ln \frac{1-p^1_{ij}}{p^1_{ij}} \,\,\, , \,\,\, llr^2_{ij} = \ln \frac{1-p^2_{ij}}{p^2_{ij}} . \end{aligned}$$
4. Perform iterative Belief-Propagation decoding^15^ for each of the 2*u* LDPC codewords of length-*n*, namely ${\bar{x}}^1_i$ and ${\bar{x}}^2_i$, for $1 \le i \le u$. The number of decoding iterations depends on a few parameters, e.g. the type of code used, the available decoding complexity and the desired decoding latency.
5. Using the LLR values obtained from the LDPC decoding, calculate the bit probability matrices values:
  15 $$\begin{aligned} p^1_{ij}=\frac{1}{\exp (llr^1_{ij})+1} \,\,\,\,\, , \,\,\,\,\, p^2_{ij}=\frac{1}{\exp (llr^2_{ij})+1} . \end{aligned}$$
6. Return to Step (2).

To summarize, the presented multi-user detection algorithm is executed at the BS side based on MAP estimation principles. This detector is not MAP estimation per definition, but an approximated MAP (due to the separate metrics computations for every user and each bit, the separate decoding of each user and the use of suboptimal LDPC decoding methods). Nevertheless, the periodic probability calculation for every bit (13) is MAP-oriented in the sense that all the collision combinations are considered according to their respective a-priori probabilities. Indeed, the simulations presented in Sec. 8 provide good results. However, the complexity associated with this type of detection is high when the rate of collisions increases. This may occur due to larger number of active users, longer error correcting codes or shorter available packet length. An efficient reduced-complexity decoding algorithm is the subject of the next section.

## Linear estimation approach

The proposed method in this section is based on finding estimates for the following modulated symbols:

16 $$\begin{aligned} {\tilde{x}}^1_{ij}=(-1)^{x^1_{ij}} \,\,\,\,\, , \,\,\,\,\, {\tilde{x}}^2_{ij}=(-1)^{x^2_{ij}} \end{aligned}$$

The estimates are based on $\vartheta$ signal values for each symbol; these values are the intercepted signals on all the receive antennas on the relevant allocated frequency bin $t_{ij}$, i.e. $F^1_{t_{ij}} , F^2_{t_{ij}} , \ldots , F^\vartheta _{t_{ij}}$.

The intercepted signal values are complex numbers, hence the problem can be formulated as an estimation of a (real) RV from a given (real) random vector of length $2 \vartheta$. Our estimator will be linear for computational feasibility (of particular significance when large antenna arrays are employed), hence statistics up to second order are required. Similar to the discussion in Sec. 5, for simplicity of notation and without loss of generality, we use the notation $\alpha = t_{ij}$, and the order of the collision occurring in bin $\alpha$ is denoted by *c*. We assume that $i=1$, and we assume that users $2 , 3 , \ldots , c$ have collided with user 1 on bin $\alpha$. We consider each estimator for ${\tilde{x}}^1_{ij}$ and ${\tilde{x}}^2_{ij}$ separately.

### Estimator for ${\tilde{x}}^1_{1j}$

The QPSK symbol transmitted by user $i=1$ on bin $\alpha$ is equal to $\mathbb {S}_{x^1_{1 j} x^2_{1 j}}$, given by (5). The probabilities required for the estimation are of the same form as (12). From (7) we get:

17 $$\begin{aligned} \mathfrak {R}(F^a_{\alpha })= & {} \sqrt{\frac{\omega }{2}} \cdot \sum _{k=1}^{c} \left\{ {\tilde{x}}^1_{k j} \cdot \mathfrak {R}(h^a_{k \alpha }) - {\tilde{x}}^2_{k j} \cdot \mathfrak {I}(h^a_{k \alpha }) \right\} + \mathfrak {R}(n_{\alpha }) , \end{aligned}$$

18 $$\begin{aligned} \mathfrak {I}(F^a_{\alpha })= & {} \sqrt{\frac{\omega }{2}} \cdot \sum _{k=1}^{c} \left\{ {\tilde{x}}^1_{k j} \cdot \mathfrak {I}(h^a_{k \alpha }) + {\tilde{x}}^2_{k j} \cdot \mathfrak {R}(h^a_{k \alpha }) \right\} + \mathfrak {I}(n_{\alpha }) . \end{aligned}$$

We shall develop an estimator for ${\tilde{x}}^1_{1j}$ given the length-$2 \vartheta$ column vector

19 $$\begin{aligned} {\bar{F}}=\left[ \mathfrak {R}(F^1_{\alpha }) \,\,\, \mathfrak {I}(F^1_{\alpha }) \,\,\, \mathfrak {R}(F^2_{\alpha }) \,\,\, \mathfrak {I}(F^2_{\alpha }) \,\,\, \ldots \,\,\, \mathfrak {R}(F^\vartheta _{\alpha }) \,\,\, \mathfrak {I}(F^\vartheta _{\alpha }) \right] ^T . \end{aligned}$$

For the estimation we shall need the covariance matrix $C_{{\bar{F}}}$, of ${\bar{F}}$; it is easy to realize that $C_{{\bar{F}}}$ consists of the following values:

20 $$\begin{aligned}&C_{{\bar{F}}}(2a-1,2z-1) = \frac{\omega }{2} \cdot \left[ \sum _{k=1}^{c} \mathfrak {R}(h^a_{k \alpha }) \cdot \mathfrak {R}(h^z_{k \alpha }) \cdot C_{{\tilde{x}}^1_{k j}} + \mathfrak {I}(h^a_{k \alpha }) \cdot \mathfrak {I}(h^z_{k \alpha }) \cdot C_{{\tilde{x}}^2_{k j}} \right] + \frac{\sigma ^2}{2} \cdot \delta (a-z) ,\end{aligned}$$

21 $$\begin{aligned}&C_{{\bar{F}}}(2a,2z) =\frac{\omega }{2} \cdot \left[ \sum _{k=1}^{c} \mathfrak {I}(h^a_{k \alpha }) \cdot \mathfrak {I}(h^z_{k \alpha }) \cdot C_{{\tilde{x}}^1_{k j}} + \mathfrak {R}(h^a_{k \alpha }) \cdot \mathfrak {R}(h^z_{k \alpha }) \cdot C_{{\tilde{x}}^2_{k j}} \right] + \frac{\sigma ^2}{2} \cdot \delta (a-z) , \end{aligned}$$

22 $$\begin{aligned}&C_{{\bar{F}}}(2a,2z-1) = \frac{\omega }{2} \cdot \left[ \sum _{k=1}^{c} \mathfrak {I}(h^a_{k \alpha }) \cdot \mathfrak {R}(h^z_{k \alpha }) \cdot C_{{\tilde{x}}^1_{k j}} - \mathfrak {R}(h^a_{k \alpha }) \cdot \mathfrak {I}(h^z_{k \alpha }) \cdot C_{{\tilde{x}}^2_{k j}} \right] , \end{aligned}$$

23 $$\begin{aligned}&C_{{\bar{F}}}(2a-1,2z) = \frac{\omega }{2} \cdot \left[ \sum _{k=1}^{c} \mathfrak {R}(h^a_{k \alpha }) \cdot \mathfrak {I}(h^z_{k \alpha }) \cdot C_{{\tilde{x}}^1_{k j}} - \mathfrak {I}(h^a_{k \alpha }) \cdot \mathfrak {R}(h^z_{k \alpha }) \cdot C_{{\tilde{x}}^2_{k j}} \right] , \end{aligned}$$

where $\delta [ \bullet ]$ is the Kronecker delta, and the variances of ${\tilde{x}}^1_{k j}$, ${\tilde{x}}^2_{k j}$ are given by:

24 $$\begin{aligned} C_{{\tilde{x}}^1_{k j}}= & {} 4 p^1_{k j} \cdot ( 1 - p^1_{k j} ) , \end{aligned}$$

25 $$\begin{aligned} C_{{\tilde{x}}^2_{k j}}= & {} 4 p^2_{k j} \cdot ( 1 - p^2_{k j} ) . \end{aligned}$$

where in (24) we take $p^1_{1j}=0.5$, rather than employing the current iteration probability value (see remark (a) on Eqn. (13)). All the other probability values are taken from the probability tables (12) that are updated periodically.

Define the following matrix $\psi _{\alpha }$ of size $2 \vartheta \times 2 c$ as follows:

26 $$\begin{aligned} \varPsi _{\alpha } = \left[ \begin{array}{ccccccc} \mathfrak {R}(h^1_{1 \alpha }) &{} \mathfrak {I}(h^1_{1 \alpha }) &{} \mathfrak {R}(h^1_{2 \alpha }) &{} \mathfrak {I}(h^1_{2 \alpha }) &{} \ldots &{} \mathfrak {R}(h^1_{c \alpha }) &{} \mathfrak {I}(h^1_{c \alpha }) \\ \mathfrak {I}(h^1_{1 \alpha }) &{} -\mathfrak {R}(h^1_{1 \alpha }) &{} \mathfrak {I}(h^1_{2 \alpha }) &{} -\mathfrak {R}(h^1_{2 \alpha }) &{} \ldots &{} \mathfrak {I}(h^1_{c \alpha }) &{} -\mathfrak {R}(h^1_{c \alpha }) \\ \mathfrak {R}(h^2_{1 \alpha }) &{} \mathfrak {I}(h^2_{1 \alpha }) &{} \mathfrak {R}(h^2_{2 \alpha }) &{} \mathfrak {I}(h^2_{2 \alpha }) &{} \ldots &{} \mathfrak {R}(h^2_{c \alpha }) &{} \mathfrak {I}(h^2_{c \alpha }) \\ \mathfrak {I}(h^2_{1 \alpha }) &{} -\mathfrak {R}(h^2_{1 \alpha }) &{} \mathfrak {I}(h^2_{2 \alpha }) &{} -\mathfrak {R}(h^2_{2 \alpha }) &{} \ldots &{} \mathfrak {I}(h^2_{c \alpha }) &{} -\mathfrak {R}(h^2_{c \alpha }) \\ \ldots &{} \ldots &{} \ldots &{} \ldots &{} \ldots &{} \ldots &{} \ldots \\ \mathfrak {R}(h^\vartheta _{1 \alpha }) &{} \mathfrak {I}(h^\vartheta _{1 \alpha }) &{} -\mathfrak {R}(h^\vartheta _{2 \alpha }) &{} \mathfrak {I}(h^\vartheta _{2 \alpha }) &{} \ldots &{} \mathfrak {R}(h^\vartheta _{c \alpha }) &{} \mathfrak {I}(h^\vartheta _{c \alpha }) \\ \mathfrak {I}(h^\vartheta _{1 \alpha }) &{} -\mathfrak {R}(h^\vartheta _{1 \alpha }) &{} \mathfrak {I}(h^\vartheta _{2 \alpha }) &{} -\mathfrak {R}(h^\vartheta _{2 \alpha }) &{} \ldots &{} \mathfrak {I}(h^\vartheta _{c \alpha }) &{} -\mathfrak {R}(h^\vartheta _{c \alpha }) \end{array} \right] , \end{aligned}$$

and the following diagonal matrix $\varLambda _{\alpha }$ of size $2 c \times 2 c$ as follows:

27 $$\begin{aligned} \varLambda _{\alpha } = diag \left( \left[ C^{\frac{1}{2}}_{{\tilde{x}}^1_{1 j}} , C^{\frac{1}{2}}_{{\tilde{x}}^2_{1 j}} , C^{\frac{1}{2}}_{{\tilde{x}}^1_{2 j}} , C^{\frac{1}{2}}_{{\tilde{x}}^2_{2 j}} , \ldots , C^{\frac{1}{2}}_{{\tilde{x}}^1_{c j}} , C^{\frac{1}{2}}_{{\tilde{x}}^2_{c j}} \right] \right) . \end{aligned}$$

Combining (20–27), we express the covariance matrix of ${\bar{F}}$ in the following form:

28 $$\begin{aligned} C_{{\bar{F}}} = \frac{\omega }{2} \cdot ( \varPsi _{\alpha } \cdot \varLambda _{\alpha } ) \cdot ( \varPsi _{\alpha } \cdot \varLambda _{\alpha } )^T + \frac{\sigma ^2}{2} \cdot {\mathcal {I}}_{2 \vartheta } , \end{aligned}$$

where ${\mathcal {I}}_{2 \vartheta }$ is the identity matrix of size $2 \vartheta$. It is easily shown that the cross-covariance vector, between ${\tilde{x}}^1_{1j}$ and ${\bar{F}}$, is equal to:

29 $$\begin{aligned} C_{{\tilde{x}}^1_{1j} {\bar{F}}} = \sqrt{\frac{\omega }{2}} \cdot [ \mathfrak {R}(h^1_{1 \alpha }) , \mathfrak {I}(h^1_{1 \alpha }) , \mathfrak {R}(h^2_{1 \alpha }) , \mathfrak {I}(h^2_{1 \alpha }) , \ldots , \mathfrak {R}(h^\vartheta _{1 \alpha }) , \mathfrak {I}(h^\vartheta _{1 \alpha }) ] . \end{aligned}$$

where again $p^1_{1j}=0.5$, hence $C_{{\tilde{x}}^1_{1 j}}=1$. For numerical calculation reasons, to be clarified later (see Obs. 1), we will express $C_{{\tilde{x}}^1_{1j} {\bar{F}}}$ in the form:

30 $$\begin{aligned} C_{{\tilde{x}}^1_{1j} {\bar{F}}} = \sqrt{\frac{\omega }{2}} \cdot \rho _1 \cdot ( \varPsi _{\alpha } \cdot \varLambda _{\alpha } )^T , \end{aligned}$$

where $\rho _1$ is a length-2*c* row vector, consisting of a single 1 and 0 elsewhere:

31 $$\begin{aligned} \rho _1 = [ 1 , 0 , 0 , \ldots , 0 ]. \end{aligned}$$

The mean of ${\bar{F}}$ is denoted by $\eta _{{\bar{F}}}$; $\eta _{{\bar{F}}}$ consists of the following values:

32 $$\begin{aligned}&\eta _{{\bar{F}}}(2a-1) = \sqrt{\frac{\omega }{2}} \cdot \sum _{k=1}^{c} \left\{ \mathfrak {R}(h^a_{k \alpha }) \cdot \eta _{{\tilde{x}}^1_{k j}} - \mathfrak {I}(h^a_{k \alpha }) \cdot \eta _{{\tilde{x}}^2_{k j}} \right\} , \end{aligned}$$

33 $$\begin{aligned}&\eta _{{\bar{F}}}(2a) = \sqrt{\frac{\omega }{2}} \cdot \sum _{k=1}^{c} \left\{ \mathfrak {I}(h^a_{k \alpha }) \cdot \eta _{{\tilde{x}}^1_{k j}} + \mathfrak {R}(h^a_{k \alpha }) \cdot \eta _{{\tilde{x}}^2_{k j}} \right\} , \end{aligned}$$

where

$$\begin{aligned} \eta _{{\tilde{x}}^1_{k j}}= & {} 1 - 2 p^1_{k j} , \\ \eta _{{\tilde{x}}^2_{k j}}= & {} 1 - 2 p^2_{k j} , \end{aligned}$$

and again $p^1_{1j}=0.5$, hence $\eta _{{\tilde{x}}^1_{1 j}}=0$.

The minimum mean-square (MMSE) linear estimator for ${\tilde{x}}^1_{1 j}$ given ${\bar{F}}$ is equal to:

34 $$\begin{aligned}&{\hat{x}}^1_{1j} = C_{{\tilde{x}}^1_{1j} {\bar{F}}} \cdot C_{{\bar{F}}}^{-1} \cdot ({\bar{F}} - \eta _{{\bar{F}}}) \nonumber \\&\quad = \sqrt{\frac{\omega }{2}} \cdot \rho _1 \cdot ( \varPsi _{\alpha } \cdot \varLambda _{\alpha } )^T \cdot \left[ \frac{\omega }{2} \cdot ( \varPsi _{\alpha } \cdot \varLambda _{\alpha } ) \cdot ( \varPsi _{\alpha } \cdot \varLambda _{\alpha } )^T + \frac{\sigma ^2}{2} \cdot {\mathcal {I}}_{2 \vartheta } \right] ^{-1} \cdot ({\bar{F}} - \eta _{{\bar{F}}}) , \end{aligned}$$

and the mean-square error of the estimator is given by

35 $$\begin{aligned} \varepsilon ^1_{1j}=E[({\tilde{x}}^1_{1j}-{\hat{x}}^1_{1j})^2] = 1 - C_{{\tilde{x}}^1_{1j} {\bar{F}}} \cdot C_{{\bar{F}}}^{-1} \cdot C^T_{{\tilde{x}}^1_{1j} {\bar{F}}} . \end{aligned}$$

The following observation has significant implications for reducing the computational complexity of the linear estimator:

#### Observation 1

$$\begin{aligned}&( \varPsi _{\alpha } \cdot \varLambda _{\alpha } )^T \cdot \left[ \frac{\omega }{2} \cdot ( \varPsi _{\alpha } \cdot \varLambda _{\alpha } ) \cdot ( \varPsi _{\alpha } \cdot \varLambda _{\alpha } )^T + \frac{\sigma ^2}{2} \cdot {\mathcal {I}}_{2 \vartheta } \right] ^{-1} \\&\quad =\left[ \frac{\omega }{2} \cdot ( \varPsi _{\alpha } \cdot \varLambda _{\alpha } )^T \cdot ( \varPsi _{\alpha } \cdot \varLambda _{\alpha } ) + \frac{\sigma ^2}{2} \cdot {\mathcal {I}}_{2 c} \right] ^{-1} \cdot ( \varPsi _{\alpha } \cdot \varLambda _{\alpha } )^T . \end{aligned}$$

Using Obs. 1, the MMSE linear estimator can be re-written in the form:

36 $$\begin{aligned} {\hat{x}}^1_{1j} = \sqrt{\frac{\omega }{2}} \cdot \rho _1 \cdot \left[ \frac{\omega }{2} \cdot ( \varPsi _{\alpha } \cdot \varLambda _{\alpha } )^T \cdot ( \varPsi _{\alpha } \cdot \varLambda _{\alpha } ) + \frac{\sigma ^2}{2} \cdot {\mathcal {I}}_{2 c} \right] ^{-1} \cdot ( \varPsi _{\alpha } \cdot \varLambda _{\alpha } )^T \cdot ({\bar{F}} - \eta _{{\bar{F}}}) . \end{aligned}$$

While the estimator in (34) requires a sized $2 \vartheta$ matrix inversion, the estimator in (36) requires a sized 2*c* matrix inversion. The number of receive antennas, $\vartheta$, is typically significantly higher than the number of users transmitting on a single bin, which is *c*. This implies that the estimator in (36) requires a significantly reduced computational complexity; more specifically, it requires an order $O(c^3)$ numerical operations, compared to an order $O(\vartheta ^3)$ numerical operations with the estimator in (34). In addition, it is more robust against accumulated calculation precision errors when using Gaussian elimination method or one of its variations for the matrix inversion.

### Estimator for ${\tilde{x}}^2_{1j}$

The derivation of an estimator for ${\tilde{x}}^2_{1j}$ is similar to the derivation for ${\tilde{x}}^1_{1j}$, herein we detail only the differences. Similar to the previous derivations, we take $p^2_{1j}=0.5$, rather than employing the current iteration probability value; all the other probability values are taken from the probability tables (12) that are updated periodically. This difference should be taken into consideration when calculating the matrix $\varLambda _{\alpha }$ (27) and the vector $\eta _{{\bar{F}}}$ (32–33).

The cross-covariance vector, between ${\tilde{x}}^2_{1j}$ and ${\bar{F}}$, is equal to:

37 $$\begin{aligned} C_{{\tilde{x}}^2_{1j} {\bar{F}}} = \sqrt{\frac{\omega }{2}} \cdot [ -\mathfrak {I}(h^1_{1 \alpha }) , \mathfrak {R}(h^1_{1 \alpha }) , -\mathfrak {I}(h^2_{1 \alpha }) , \mathfrak {R}(h^2_{1 \alpha }) , \ldots , -\mathfrak {I}(h^\vartheta _{1 \alpha }) , \mathfrak {R}(h^\vartheta _{1 \alpha }) ] . \end{aligned}$$

Similar to (30), we express $C_{{\tilde{x}}^2_{1j} {\bar{F}}}$ in the form:

38 $$\begin{aligned} C_{{\tilde{x}}^2_{1j} {\bar{F}}} = \sqrt{\frac{\omega }{2}} \cdot \rho _2 \cdot ( \varPsi _{\alpha } \cdot \varLambda _{\alpha } )^T , \end{aligned}$$

where $\rho$ is a length-2*c* row vector, consisting of a single -1 and 0 elsewhere:

39 $$\begin{aligned} \rho _2 = [ 0 , -1 , 0 , \ldots , 0 ]. \end{aligned}$$

### Linear estimation: description of the detection algorithm

Using the estimators for ${\tilde{x}}^1_{1j}$ and ${\tilde{x}}^2_{1j}$ above, the complete detection algorithm can be formulated as follows:

#### Algorithm 2

1. Initialize the probability matrices $p^1_{ij}=p^2_{ij}=0.5$, $1 \le i \le u$, $1 \le j \le n$.
2. Calculate all the linear estimators ${\hat{x}}^1_{ij}$, ${\hat{x}}^2_{ij}$ and their corresponding mean-square errors $\varepsilon ^1_{ij}$, $\varepsilon ^2_{ij}$ using the results in subsec. 6.1 and 6.2.
3. Compute the log-likelihood ratio (Note that this algorithmic ratio calculation assumes that ${\hat{x}}^1_{ij}$ and ${\hat{x}}^2_{ij}$ have Gaussian distribution; an assumption which is not always accurate. However, it is shown, via simulation, to provide a very good approximation) for each bit of every user:
  40 $$\begin{aligned} llr^1_{ij}=-\frac{1}{2 \varepsilon ^1_{ij}} [({\hat{x}}^1_{ij}-1)^2-({\hat{x}}^1_{ij}+1)^2] \,\,\,\, , \,\,\,\, llr^2_{ij}=-\frac{1}{2 \varepsilon ^2_{ij}} [({\hat{x}}^2_{ij}-1)^2-({\hat{x}}^2_{ij}+1)^2]. \end{aligned}$$
4. Run iterative Belief-Propagation (BP) decoding^15^ for each of the 2*u* length-*n* LDPC codewords, namely ${\bar{x}}^1_i$ and ${\bar{x}}^2_i$, for $1 \le i \le u$.
5. Using the LLR values obtained from the LDPC decoding, calculate the bit probability matrices values (see (15)).
6. Return to step (2).

To summarize, we presented a detection algorithm carried out at the BS, which is based on a linear estimator, combined with an LDPC decoding method. This detection scheme has a low decoding complexity due to the simplified (linear) processing of collision events. The scheme is readily implementable for practical MU-MIMO and OFDRMA schemes, at the price of somewhat degraded error rate performance. Simulation results are presented in Sec. 8.

## Uplink OFDRMA capacity

Each user transmits towards the BS on any of the frequency bins included in its allocation vector (Def. 1). Let us focus on the $i^{th}$ user. From this user’s perspective, any transmission on any of its allocated bins $t_{ij}$, $1 \le j \le n$, can be corrupted by an interference of between 0 (no-interference) to $u-1$ (maximum-interference) other users. The received signals at the base station, on any allocated bin $\alpha = t_{ij}$, can be re-written using (7) in the form:

41 $$\begin{aligned} {\bar{F}}_{\alpha } = H_{i \alpha } \cdot X_{ij} + \sum _{{\tilde{i}}=1, {\tilde{i}} \ne i}^{u} {\tilde{H}}_{{\tilde{i}} \alpha } \cdot X_{{\tilde{i}} {\tilde{j}}} + {\bar{n}}_{\alpha } , \end{aligned}$$

where ${\bar{F}}_{\alpha }$ is a length-$\vartheta$ column vector containing all the received signals on all the BS receive antennas, i.e., $F^a_{\alpha }$ for $1 \le a \le \vartheta$. Similarly, ${\bar{n}}_{\alpha }$ is a length-$\vartheta$ column vector containing all the noise samples on all the BS receive antennas, i.e., $n^a_{\alpha }$ for $1 \le a \le \vartheta$. $H_{i \alpha }$ is a length-$\vartheta$ column vector containing all the path gains between the $i^{th}$ user and all the receive antennas, i.e., $h_{i \alpha }^a$ for $1 \le a \le \vartheta$. Each ${\tilde{H}}_{{\tilde{i}} \alpha }$ is a length-$\vartheta$ column vector with a similar construct to $H_{i \alpha }$, with the following unique statistical property:

42 $$\begin{aligned} {\tilde{H}}_{{\tilde{i}} \alpha } = \left\{ \begin{array}{c} \,\,\,\,\,\,\, [0 \, 0 \, \ldots \, 0]^T \,\,\,\,\,\,\,\,\,\,\,\,\,\,\,\,\,\,\,\,\,\,\,\,\, w.p. \,\,\,\, 1-p \, , \\ \!\!\!\!\!\!\!\! \left[ h_{{\tilde{i}} \alpha }^1 \, h_{{\tilde{i}} \alpha }^2 \, \ldots \, h_{{\tilde{i}} \alpha }^\vartheta \right] ^T \,\,\,\,\,\,\,\, w.p. \,\,\,\, p \, , \end{array} \right. \end{aligned}$$

where *p* is the single user Bernoulli distribution probability, as discussed in Sec. 3. The probability for ${\tilde{H}}_{{\tilde{i}} \alpha }$ to have non all-zeroes value is equal to the probability to have a collision order of 1 on bin $\alpha$, as detailed in Sec. 3. Note that the form in (41) is not mathematically identical to the form in (7), as (41) has independent randomness in each and every separate bin, while (7) has randomness in the allocation of frequency bins over the entire OFDM packet. The form (41–42) is however correct from the statistical point of view, hence relevant for the capacity analysis that follows. The entries in $H_{i \alpha }$ and ${\tilde{H}}_{{\tilde{i}} \alpha }$ are independent, which comes from the independency of the path gains $h_{i \alpha }^a$ and the independent nature of the allocations between any user to the others. We assume all users employ Gaussian code books, the usual form of optimum signal in MIMO problems^17,18^. Then, if we condition on ${\tilde{H}}_{{\tilde{i}} \alpha }$, ${\tilde{i}} = 1 \ldots u$, ${\tilde{i}} \ne i$, the interference-plus-noise from (41), $\sum _{{\tilde{i}}=1, {\tilde{i}} \ne i}^{u} {\tilde{H}}_{{\tilde{i}} \alpha } \cdot X_{{\tilde{i}} {\tilde{j}}} + {\bar{n}}_{\alpha }$, is Gaussian distributed with covariance matrix:

43 $$\begin{aligned} C_i = \sum _{{\tilde{i}}=1, {\tilde{i}} \ne i}^{u} {\tilde{H}}_{{\tilde{i}} \alpha } \cdot \omega \cdot {\tilde{H}}_{{\tilde{i}} \alpha }^H + \sigma ^2 \cdot {\mathcal {I}}_{\vartheta } , \end{aligned}$$

where $\omega$ is the mean signal energy, similar to (6). As the base station has knowledge of the channel path gains and the users allocations, the interference-plus-noise is whitened by multiplying ${\bar{F}}_{\alpha }$ by $C_i^{\frac{1}{2}}$. Using similar results from previous work^19^, the mutual information between the $i^{th}$ user input and the BS output can be expressed in the form:

44 $$\begin{aligned}&I(X_{ij} ; {\bar{F}}_{\alpha }, {\mathcal {H}}) = E \left\{ \log _2 \left[ \det \left( {\mathcal {I}}_{\vartheta } + \omega \cdot H_{i \alpha } \cdot H_{i \alpha }^H \cdot C_i^{-1} \right) \right] \right\} \nonumber \\&\quad = E \left\{ \log _2 \left[ 1 + \omega \cdot H_{i \alpha }^H \cdot C_i^{-1} \cdot H_{i \alpha } \right] \right\} \nonumber \\&\quad = E \left\{ \log _2 \left[ 1 + \frac{\omega }{\sigma ^2} \cdot H_{i \alpha }^H \cdot \left( \frac{C_i}{\sigma ^2} \right) ^{-1} \cdot H_{i \alpha } \right] \right\} , \end{aligned}$$

where ${\mathcal {H}}$ denotes the complete channel path gain information available to the BS, and the identity $\det (I + AB) = \det (I +BA)$ was used.

It would be useful, in addition, to compute the mutual information strictly for a specific given collision order on bin $\alpha$. We will denote this by $I_c$ for shortage of notation:

45 $$\begin{aligned} I_c = I(X_{ij} ; {\bar{F}}_{\alpha }, {\mathcal {H}} | c_{\alpha } = c) . \end{aligned}$$

Where $I_0 = 0$ refers to an unused bin whose mutual information is null. Unfortunately, in general, there are no closed form expressions for each $I_c$, and they have to be calculated numerically by Monte Carlo simulations. It is easy to see that every $I_c$ depends only on the number of receive antennas and the SNR ratio $\frac{\omega }{\sigma ^2}$.

Keeping in mind that the base station knows all users bin allocations and hence the collision order $c_{\alpha }$ on each bin $\alpha$, and that a bin with collision order $c_{\alpha } = c$ serves *c* users on bin $\alpha$ simultaneously, the average mutual information per-bin per-user is calculated as follows:

46 $$\begin{aligned} {\bar{I}} = \frac{1}{u} \cdot \sum _{c=0}^{c=u} p(c_{\alpha } =c) \cdot c \cdot I_c = \frac{1}{u} \cdot \sum _{c=0}^{c=u} c \cdot \left( {\begin{array}{c}u\\ c\end{array}}\right) \cdot p^c \cdot (1-p)^{u-c} \cdot I_c , \end{aligned}$$

where $p(c_{\alpha } =c)$ is the binomial distribution taken from (9). *p* is the probability (8) for a single user to occupy bin $\alpha$, equal to the ratio between the code length to the OFDM packet length, i.e., $\frac{n}{m}$. Naturally, when discussing channel capacity, the code length *n* is very large, and therefore necessarily the packet length *m* is also very large, as $m \ge n$. However, in terms of maximizing capacity, *n* should not necessarily take the maximum possible value, which is *m*. We would like to maximize (46) as a function of *p*. Differentiating (46) with respect to *p*, we get:

47 $$\begin{aligned} \frac{d {\bar{I}}}{d p} = \frac{1}{u} \cdot \sum _{c=0}^{c=u} c \cdot \left( {\begin{array}{c}u\\ c\end{array}}\right) \cdot \left[ c \cdot p^{c-1} \cdot (1-p)^{u-c} - (u-c) \cdot p^c \cdot (1-p)^{u-c-1} \right] \cdot I_c . \end{aligned}$$

${\bar{I}}$ is a polynomial in *p* with order *u*, therefore $\frac{d {\bar{I}}}{d p}$ is a polynomial with order $u-1$. Therefore, equating (47) to zero yields, in general, $u-1$ solutions. As *p* takes values strictly in the range $0 < p \le 1$, only solutions in this range should be considered. In addition, $p=1$ should be considered as a candidate for maximizing ${\bar{I}}$ if $\frac{d {\bar{I}}}{d p} (p=1) > 0$. It is easy to see that:

48 $$\begin{aligned} \frac{d {\bar{I}}}{d p} (p=1) = u \cdot (I_u - I_{u-1}) + I_{u-1} , \end{aligned}$$

meaning that if

49 $$\begin{aligned} u \cdot I_u > (u-1) I_{u-1} , \end{aligned}$$

$p=1$ should also be considered.

The Abel-Ruffini theorem states that no closed-form solutions exist for polynomial equations of degree 5 or higher, therefore when the number of active users *u* is 6 or higher, no closed-form solution exists for $\frac{d {\bar{I}}}{d p} =0$, and the optimal *p* values must be calculated numerically.

A more strict scenario than (49) can be useful as follows:

### Observation 2

If

50 $$\begin{aligned} u \cdot I_u > (i-1) \cdot I_{i-1} \end{aligned}$$

for every $2 \le i \le u$, then ${\bar{I}}$ is maximized by setting $p=1$.

### Proof

Let $p_c$, $0 \le p_c \le 1$, be the probability for a collision of order *c* on bin $\alpha$, i.e. $p(c_{\alpha } = c) = p_c$. We would optimize the solution for the ’global’ problem, i.e. maximize ${\bar{I}}$ when $p_c$ are not limited to binomial distribution, as long as $\sum _{c=0}^{c=u} p_c =1$. Using the first equality in (46) and setting $p_u=1-\sum _{c=0}^{c=u-1} p_c$, we differentiate ${\bar{I}}$ by each $p_c$, $1 \le c \le u-1$:

51 $$\begin{aligned} \frac{d {\bar{I}}}{d p_c} = \frac{c}{u} \cdot I_c - I_u . \end{aligned}$$

Combining (51) with (50), we get that $\frac{d {\bar{I}}}{d p_c} < 0$ for all $1 \le c \le u-1$ (where $\frac{d {\bar{I}}}{d p_0} < 0$ always). This implies that in order to maximize ${\bar{I}}$, the values of $p_c$ for $1 \le c \le u-1$ should take the lowest value possible, which is 0; therefore $p_u=1$. Now, note that this ’global’ optimization solution coincides with the specific binomial distribution for $p=1$. Therefore $p=1$ maximizes ${\bar{I}}$ for the binomial distribution. $\square$

### Example: $u=3$

We compute the explicit closed-form solutions for *p* when the number of active users is $u=3$. From (47) we get:

52 $$\begin{aligned} {\bar{I}} = (I_1 - 2 I_2 + I_3) \cdot p^3 + 2 \cdot (-I_1 + I_2) \cdot p^2 + I_1 \cdot p . \end{aligned}$$

The first derivative is equal to:

53 $$\begin{aligned} \frac{d {\bar{I}}}{d p} = 3 \cdot (I_1 - 2 I_2 + I_3) \cdot p^2 + 4 \cdot (-I_1 + I_2) \cdot p + I_1 , \end{aligned}$$

and the second derivative is equal to:

54 $$\begin{aligned} \frac{d^2 {\bar{I}}}{d p^2} = 6 \cdot (I_1 - 2 I_2 + I_3) \cdot p + 4 \cdot (-I_1 + I_2) . \end{aligned}$$

Solving $\frac{d {\bar{I}}}{d p} =0$, we get:

55 $$\begin{aligned} p_{1,2} = \frac{2 \cdot (I_1 -I_2) \pm \sqrt{4 \cdot (I_1 -I_2)^2 - 3 \cdot (I_1 -2 I_2 +I_3) \cdot I_1}}{3 \cdot (I_1 - 2 I_2 + I_3)} . \end{aligned}$$

Keeping in mind that ${\bar{I}}(p=0) = 0$ and that ${\bar{I}} > 0$ for $0 < p \le 1$, we implement the following optimization steps for the specific scenario of $u=3$:

#### Algorithm 3

1. If Obs. 2 holds, $p=1$ is the optimum. If not, continue.
2. If $4 \cdot (I_1 -I_2)^2 - 3 \cdot (I_1 -2 I_2 +I_3) \cdot I_1 <0$, $p=1$ is the optimum. If not, continue.
3. If $p_1$, $p_2$ are both outside the range $[0 - 1]$, $p=1$ is the optimum. If not, continue.
4. For $i=1,2$, if $0< p_i < 1$ and $\frac{d^2 {\bar{I}}}{d p^2}(p=p_i) < 0$, add $p = p_i$ as a candidate.
5. If $3 I_3 > 2 I_2$, add $p=1$ as a candidate.
6. If there are 2 candidates, choose the one that gives the larger ${\bar{I}}$.

### Capacity of MU-MIMO

Finally, we would like to compare the capacity achieved in OFDRMA with the capacity achieved in the standard approach, namely the MU-MIMO scheme. In the context of this work, the common characteristic of both approaches is that both are non-orthogonal, in the sense that several users may occupy the same subcarrier simultaneously (OFDMA). The difference between the two is that in MU-MIMO a controller is needed to orchestrate and synchronize the active users, aiming at achieving (as close as possible) uniform distribution of subcarriers occupancy. We will derive the expression for the capacity in the case of MU-MIMO, similar to our previous analysis in OFDRMA. In the context of the following analysis, we denote by *p* the ratio between the code length and the packet length, i.e., $p=\frac{n}{m}$ again, but this time *p* does not have the same meaning as a parameter in the Bernoulli and Binomial distributions. The controller aims at creating a uniform distribution of subcarriers, meaning that each subcarrier has a collision order of either $\lfloor {p \cdot u} \rfloor$ or $\lfloor {p \cdot u} \rfloor + 1$. The probabilities for each collision order are $p(c_{\alpha } = \lfloor {p \cdot u} \rfloor )$ and $p(c_{\alpha } = \lfloor {p \cdot u} \rfloor +1)$, respectively, where

56 $$\begin{aligned} p(c_{\alpha } = \lfloor {p \cdot u} \rfloor ) + p(c_{\alpha } = \lfloor {p \cdot u} \rfloor + 1) = 1. \end{aligned}$$

The average collision order must be $p \cdot u$, therefore:

57 $$\begin{aligned} p(c_{\alpha } = \lfloor {p \cdot u} \rfloor ) \cdot \lfloor {p \cdot u} \rfloor + p(c_{\alpha } = \lfloor {p \cdot u} \rfloor +1) \cdot (\lfloor {p \cdot u} \rfloor +1) = p \cdot u . \end{aligned}$$

From (56) and (57), we get:

58 $$\begin{aligned} p(c_{\alpha } = \lfloor {p \cdot u} \rfloor ) = \lfloor {p \cdot u} \rfloor + 1 - p \cdot u \,\,\,\,\, , \,\,\,\,\, p(c_{\alpha } = \lfloor {p \cdot u} \rfloor + 1) = p \cdot u - \lfloor {p \cdot u} \rfloor . \end{aligned}$$

The average mutual information per-bin per-user for all the users, denoted by ${\bar{I}}_M$ for MU-MIMO, is calculated as follows:

59 $$\begin{aligned}&{\bar{I}}_M = \frac{1}{u} \cdot \left\{ p(c_{\alpha } = \lfloor {p \cdot u} \rfloor ) \cdot \lfloor {p \cdot u} \rfloor \cdot I_{\lfloor {p \cdot u} \rfloor } \right. \nonumber \\&\qquad \left. + p(c_{\alpha } = \lfloor {p \cdot u} \rfloor +1) \cdot (\lfloor {p \cdot u} \rfloor +1) \cdot I_{\lfloor {p \cdot u} \rfloor +1} \right\} . \end{aligned}$$

#### Observation 3

In order to maximize ${\bar{I}}_M$, *m* should be a multiple of *u*, i.e. $m = q \cdot u$, for an integer positive *q* (typically $q \gg 1$ as we aim at achieving capacity). The maximum for ${\bar{I}}_M$ will be achieved by choosing one of the following values for *n*: $\left[ q , 2q , \ldots , (u-1) \cdot q , u \cdot q \right]$, s.t. *p* gets one of the following values: $\frac{1}{u} , \frac{2}{u} , \ldots , \frac{u-1}{u} , 1$.

#### Proof

First, assume that $p \ne \frac{1}{u} , \frac{2}{u} , \ldots , 1$. Then, the value of $\lfloor {p \cdot u} \rfloor$ is fixed when differentiating ${\bar{I}}_M$ by *p*:

60 $$\begin{aligned} \frac{d {\bar{I}}_M}{d p} = - \lfloor {p \cdot u} \rfloor \cdot I_{\lfloor {p \cdot u} \rfloor } + (\lfloor {p \cdot u} \rfloor +1) \cdot I_{\lfloor {p \cdot u} \rfloor +1} . \end{aligned}$$

We can see from (60) that the sign (and value) of $\frac{d {\bar{I}}_M}{d p}$ remain fixed for *p* in the range $\frac{s}{u}< p < \frac{s+1}{u}$ for any integer *s*, $0 \le s \le u-2$. Therefore, a local maximum for ${\bar{I}}_M$ occurs if and only if $p=\frac{s+1}{u}$, $-s \cdot I_s + (s+1) \cdot I_{s+1} > 0$, and $-(s+1) \cdot I_{s+1} + (s+2) \cdot I_{s+2} <0$. The collection of the local maxima is the group of candidates, and the absolute maximum is chosen as the candidate that gives the largest ${\bar{I}}_M$. In addition, $p=1$ is also a candidate if $-(u-1) \cdot I_{u-1} + u \cdot I_u > 0$ (note that this is the same condition for $p=1$ as in the OFDRMA optimization). The maximum value of $I_M$ is therefore equal to:

$$\begin{aligned} \frac{1}{u} \cdot \max \left[ I_1 , 2 I_2 , \ldots , (u-1) \cdot I_{u-1} , u \cdot I_u \right] . \end{aligned}$$

$$\square $$

**Figure 2:**
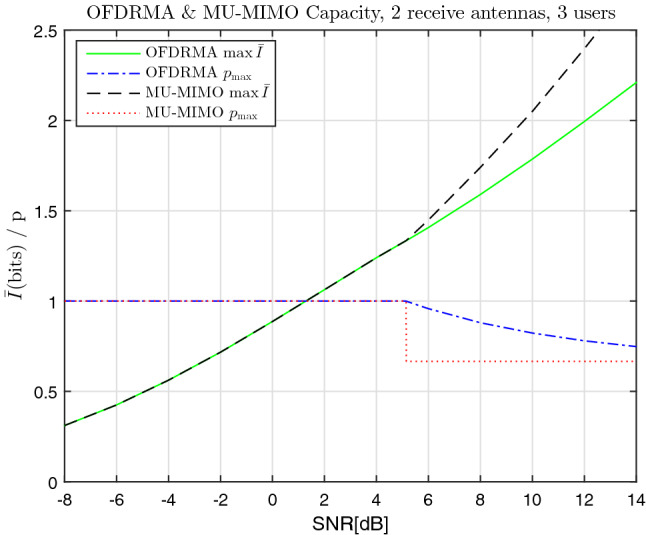
OFDRMA & MU-MIMO comparison of capacity and corresponding *p* values vs SNR, $\vartheta =2$, $u =3$.

**Figure 3:**
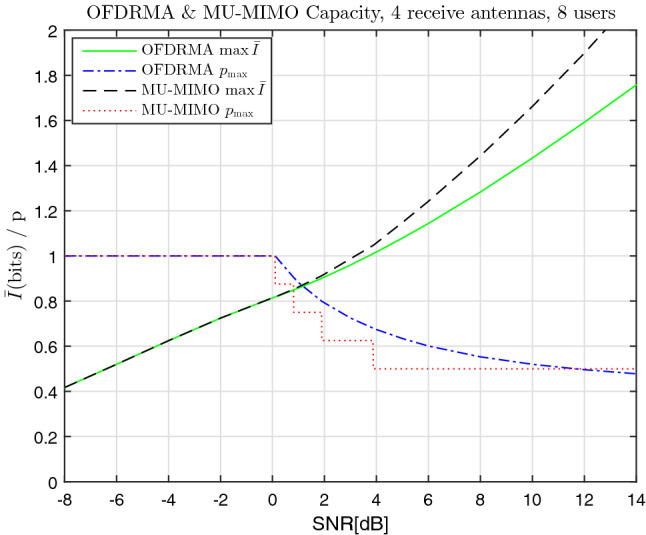
OFDRMA & MU-MIMO comparison of capacity and corresponding *p* values vs SNR, $\vartheta =4$, $u =8$.

Capacity curves as a function of SNR for $\vartheta =2$ transmit antennas and $u =3$ users are given in Fig. 2. The values of *p* that provide these maximum-entropy values are also displayed on the same graphs, denoted by $p_{max}$. Both OFDRMA and MU-MIMO results are given in each graph for comparison, using Alg. 3 and Obs. 3, respectively. Similarly, capacity curves for $\vartheta =4$ transmit antennas and $u =8$ users are given in Fig. 3, with the exception that the larger number of users implies that numerical maximization of the entropy was required for OFDRMA, as explained after (49).

We can see that for low SNR values the optimal *p* values are equal to $p_{max} =1$. This is evident in Fig. 2 for $SNR \le 5.14 \, dB$ and in Fig. 3 for $SNR \le 0.1 \, dB$. This is explained by that the additive Gaussian noise is more significant compared to the multi-users interference, therefore it is beneficial to use all the available bandwidth for each user to combat the stronger additive noise. In this low-SNR region the capacity outputs of the OFDRMA and MU-MIMO coincide. Above a certain SNR ratio (depending on the values of $\vartheta$ and *u*), this scenario gradually changes, with $p_{max}$ values decreasing, and an increasing gap in the capacity values is observed in favor of MU-MIMO over OFDRMA. However, as we shall see in the following simulation section, in the SNR region corresponding to practical bit error rates, this gap is not significant.

**Figure 4:**
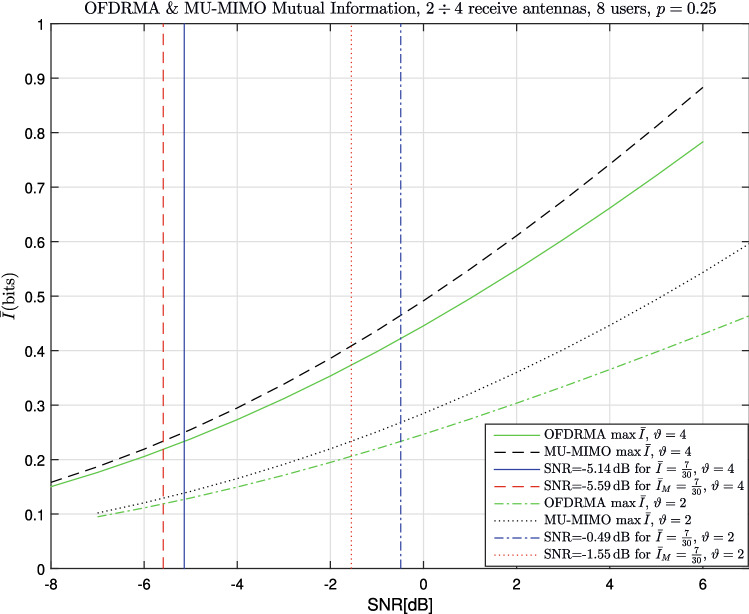
OFDRMA & MU-MIMO comparison of Mutual Information vs SNR, $p=0.25$, $\vartheta =2 \div 4$, $u =8$.

In addition, mutual information curves for a fixed *p* value are provided for $u=8$ with $\vartheta =4$, and $u=8$ with $\vartheta =2$. This type of analysis can be useful in practical systems where the code length *n* and the packet length *m* are strictly pre-defined, hence the ratio $p=\frac{n}{m}$ is fixed. The results are given in Fig. 4 for $p=0.25$. Also displayed in the graphs are 4 vertical lines, which represent the SNR values required to achieve ${\bar{I}} = \frac{7}{30}$ and ${\bar{I}}_M = \frac{7}{30}$, for both $\vartheta =4$ and $\vartheta =2$. The choices of the values $p=0.25$ and mutual information of $\frac{7}{30}$ will be clarified along with the simulation results in Sec. 8, where the simulations layout used the parameter $p=0.25$ and the coding rate per-user per-bin is equal to $\frac{7}{30}$. The ’gap’ between the SNR required to achieve ${\bar{I}} = \frac{7}{30}$ and ${\bar{I}}_M = \frac{7}{30}$ is equal to $0.45 \, dB$ for $\vartheta =4$ and $1.06 \, dB$ for $\vartheta =2$. This gap is naturally in favor of the traditional MU-MIMO scheme over the novel OFDRMA scheme. Further comparisons of the results in Fig. 4 and the simulation results are included in Sec. 8.

## Simulation results

Extensive simulations were carried out using LDPC code of length $n=120$ and dimension $k=56$. This code has a sparse parity check matrix with constant column order of 3, and irregular row order of 5 or 6, and has no 4-cycles in its graph. This code is labeled 120.64.3.109 in the Encyclopedia of Sparse Graph Codes^16^. The rest of the simulation parameters, i.e., the number of active users, number of receive antennas and packet length were chosen to represent a wide variety of scenarios for our proposed uplink scheme, and to demonstrate the robustness of OFDRMA against the forced randomness induced in the system.

OFDM packet length is equal to 4 times the code length, namely $m=480$, which implies that $p= \frac{n}{m} =0.25$. QPSK modulation was used in all simulations. The number of users ranges between $u=1$ to $u=8$, where 8 users implies that the number of users in each bin (10) is equal to $u \cdot p = 2$ on the average for OFDRMA, and is exactly 2 in each bin for MU-MIMO. The coding rate per-user per-bin is equal to $\frac{2 \cdot k}{m} = \frac{7}{30}$, which explains the SNR comparisons for this specific mutual information on Fig. 4.

**Figure 5:**
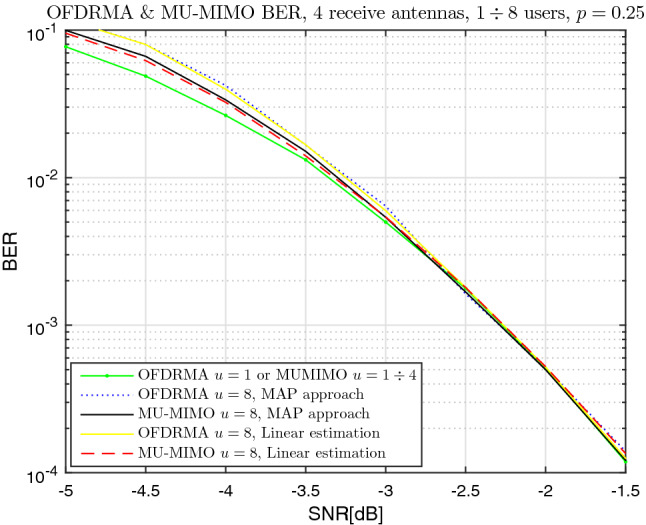
BER vs SNR, OFDRMA & MU-MIMO, QPSK, $\vartheta =4$, $u=1 \div 8$, $p=0.25$.

Fig. 5 presents the bit error rate (BER) for $\vartheta =4$ receive antennas, for both the OFDRMA and MU-MIMO methods. For each method, both the MAP approach, detailed in Sec. 5, and the linear estimation approach, detailed in Sec. 6, are displayed. Note that with MU-MIMO, the performance for users number of between $u=1$ to $u=4$ remains the same; this comes as a result of the choice $m=4 \cdot n$, therefore there are no collisions in the range $1 \le u \le 4$ for MU-MIMO.

The degradation of the OFDRMA scheme compared to the state of the art MU-MIMO is between 0.25dB (for higher BER) to 0dB (for smaller BER) for $u=8$ users. This is smaller than the theoretical mutual information gap shown in Fig. 4 (to our satisfaction). Few factors affect the difference between the theoretical and practical results, in particular the relatively short code length *n* and the sub-optimality of the decoding scheme.

The difference in the simulation results between the MAP and linear estimation approaches is found to be negligible for both OFDRMA and MU-MIMO.

In terms of the effect of number of users on performance, when *u* is increased from $u=1$ to $u=8$, the degradation is between 0.5dB to 0dB for MU-MIMO, and between 0.75dB to 0dB for OFDRMA; this gap in SNR becomes smaller for decreasing BER values.

**Figure 6:**
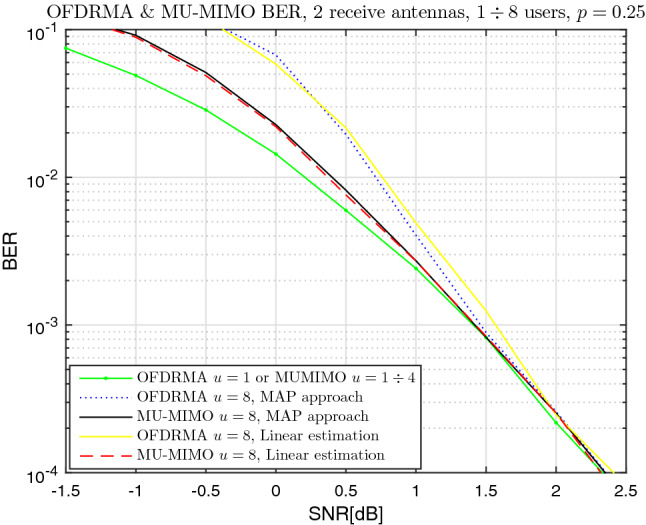
BER vs SNR, OFDRMA & MU-MIMO, QPSK, $\vartheta =2$, $u=1 \div 8$, $p=0.25$.

Similarly, Fig. 6 presents the BER for $\vartheta =2$ receive antennas. The degradation of the OFDRMA scheme compared to the state of the art MU-MIMO is between 0.8dB (for higher BER) to 0dB (for smaller BER) for $u=8$ users. Again, this is smaller than the theoretical mutual information gap shown in Fig. 4, in correspondence with the explanation above. Evidently, the degradation of the OFDRMA scheme compared to the state of the art MU-MIMO is more significant with $\vartheta =2$ compared to $\vartheta =4$. This is explained by that for OFDRMA, some bins can have high collision order (compared to the uniform distribution of MU-MIMO) due to the random allocation of users to bins. These bins transfer information poorly when the number of receive antennae is smaller, as the received diversity may be insufficient to cope with the higher collision orders on these bins.

The difference in the simulation results between the MAP and linear estimation approaches is negligible for MU-MIMO; for OFDRMA, the gap is found to be between 0dB to 0.1dB.

With respect to the effect of the number of users on performance, when *u* is increased from $u=1$ to $u=8$, the degradation is between 0.7dB to 0dB for MU-MIMO, and between 1.5dB to 0.05dB for OFDRMA. The detrimental effect of the increased number of users on performance is greater with $\vartheta =2$ compared to $\vartheta =4$. This is explained by the fact that as the number of users increases, so does the collisions order. This is more difficult to handle at the BS side when the number of receive antennas, hence the received diversity order, is smaller.

## Conclusions

OFDM with random multiple access (OFDRMA) was presented for uplink transmission. The capacity of the scheme was analyzed comprehensively and compared with the capacity of a comparable state of the art scheme, namely MU-MIMO. Two detection schemes, which vary in complexity, were described. These schemes were shown via simulation to provide good performance and robustness against the forced randomness of the system. The practical degradation in the performance of OFDRMA with respect to MU-MIMO was found to be actually less than that predicted by the theoretical capacity analysis. Nowadays, with the emergence of MU-MIMO and IoT related standards, relaying on NOMA in OFDMA is called for. The OFDRMA approach may prove as a viable alternative to MU-MIMO in scenarios where uplink communications cannot rely on BS oriented downlink messages that orchestrate the frequency resources allocations.
